# Minimizing the Risk of Diagnostic Errors in Acute Care for Older Adults: An Interdisciplinary Patient Safety Challenge

**DOI:** 10.3390/healthcare12181842

**Published:** 2024-09-13

**Authors:** Baker Nawfal Jawad, Kirstine Zink Pedersen, Ove Andersen, Ninna Meier

**Affiliations:** 1 Department of Clinical Research, Copenhagen University Hospital Amager and Hvidovre, Hvidovre, 2650 Copenhagen, Denmark; ove.andersen@regionh.dk; 2 Department of Clinical Medicine, University of Copenhagen, 2200 Copenhagen, Denmark; 3 Department of Organization, Copenhagen Business School, 2000 Frederiksberg, Denmark; kzp.ioa@cbs.dk; 4 Emergency Department, Copenhagen University Hospital Amager and Hvidovre, 2650 Hvidovre, Denmark; 5 Department of Sociology and Social Work, Aalborg University, 9220 Aalborg, Denmark; meier@socsci.aau.dk

**Keywords:** diagnostic errors, clinical decision-making, acute care, interdisciplinary research

## Abstract

Modern healthcare systems are increasingly organized according to diagnosis-specific clinical pathways and treatment protocols. At the same time, the number of patients with complex problems and needs that do not fit the single-diagnosis approach is rising, contributing to a high prevalence of diagnostic errors. In this article, we focus on the risk of diagnostic errors arising from missed or incomplete diagnosis and assessment of older adult patients’ care needs in the first hours of acute hospitalizations in EDs. This focus is important for improving patient safety, as clinical decisions made in EDs impact patient safety in the subsequent steps of the process, thereby potentially causing new risks to arise. Based on our discussion of clinical decision-making and diagnostic errors in the acute care context, we propose a more comprehensive interdisciplinary approach to improvements in patient safety that integrates organizational and clinical research and examines where, when, how, and why risks to patient safety arise in and across different clinical–organizational contexts.

## 1. Introduction

Paradoxically, while healthcare systems are increasingly organized according to diagnosis-specific clinical pathway protocols, the number of patients with more complex problems that do not fit the single-diagnosis approach is increasing. Older adults with chronic diseases, both coexisting diseases (multimorbidity) or occurrences of distinct additional diseases (comorbidity) [1], are the fastest-growing population in acute care and emergency departments (ED). This group represents more than half of the total hospitalizations [2] and is associated with individual and organizational patient safety concerns. The diagnosis and treatment of such patients are further complicated by atypical presentations of signs and symptoms, which heightens clinical uncertainty. Previous research indicates that older adults are particularly vulnerable to missed diagnoses. For instance, 12.5% of older adults hospitalized from emergency departments had significant discrepancies between their admission and discharge diagnoses [3,4].

Diagnostic errors can have serious consequences for individuals, healthcare organizations, healthcare systems, and societies, yet we still lack systematic and reliable strategies for investigating and improving clinical decision-making and its impact on patient safety [5]. Diagnostic errors are both common and harmful to patient safety [5], and they have been defined as *a failure to (a) establish an accurate and timely explanation of the patient’s health problem or (b) communicate that explanation to the patient* [6] (p.85). The best available evidence indicates that cognitive errors and failures in judgment dominate. However, diagnostic errors are more complex events than an individual’s clinical decision [7]. Diagnostic errors occur in clinical decision-making processes, which we understand to be influenced by three main elements: i: the content (knowledge/data), ii: the decision-making practices (interactions between the patient, health professional, and decision-support tools, e.g., information technologies), and iii: the context in which this takes place (for instance, organizational and national). Thus, several factors influence where, when, and how risks arise in diagnostic work, because diagnostic errors are influenced by various other factors, like system dynamics, patient characteristics, and staff interactions, and present a continuous challenge in healthcare [8]. These errors raise important questions about designing and implementing effective measures for improvement across healthcare organizations. Unaddressed diagnostic errors can quickly lead to harm, either directly or through less apparent harmful events. This is especially important for patients with complex conditions, where such errors often result in repeated hospitalizations, mistreatment, and increased stress for both the patients and healthcare organizations.

The aim of this opinion article is to discuss diagnostic errors, specifically the risks that arise from missed, untimely, or incomplete diagnoses and assessments of complex care needs in older adult patients during the initial hours of acute hospitalizations in EDs, separate from urgent triage. However, the lack of consensus on defining diagnostic errors complicates any quantification efforts, necessitating an interdisciplinary approach to thoroughly conceptualize the problem before investigating its full extent [9].

Positioned in the context of acute hospitalizations, we reflect on the risks of diagnostic errors that are more broadly relevant to the management of and improvement in patient safety issues, looking beyond the symptom-based approach of primary care and the diagnosis-based approach of secondary care and into the merging of these two approaches in acute care. We believe that the patient safety problem pertaining to risk of diagnostic errors is both clinical and organizational in nature and thus requires an interdisciplinary approach to patient safety research and improvement.

Research indicates that the characteristics of an illness strongly influence the likelihood of diagnostic errors. Studies have shown that specific symptoms generally lead to more accurate diagnoses, while more subtle ones can result in higher rates of errors [10]. In acute care settings, where older patients often present with complex yet subtle symptoms, the risk of diagnostic errors increases significantly during the initial hours of admission. This risk is further amplified when non-specific complaints (NSCs), which typically involve poorly defined symptoms with low discriminatory power, are present. Atypical or NSCs have been consistently identified as the strongest predictors of diagnostic error across various diseases [7,10]. Common atypical or NSC presentations in older patients, such as ‘fatigue,’ ‘headaches,’ ‘dizziness,’ or ‘dehydration,’ often lead to misdiagnoses. For instance, these symptoms might be mistaken for less severe conditions but could actually indicate more serious issues, such as a stroke [7,10]. This specific challenge of diagnostic errors in older patients underlines a larger systemic issue. The combination of an aging population with complex care needs, economic and time pressures, fragmented primary and secondary healthcare systems, and staffing issues can be understood as *‘messy objects’* [11]. These factors collectively challenge and pose threats to patient safety. Despite these complexities, the dominant patient safety methodologies are primarily developed to deal with tangible, identifiable, and easily fixable safety problems, often neglecting the intricacies of complex diagnostic processes [12,13,14]. This is surprising given the scale, spread, and complexity of the abovementioned problems and challenges. We believe that these pervasive and complex patient safety challenges call for interdisciplinary approaches that incorporate management, organization, and clinical practice in recognition of the importance of systemic and organizational factors in diagnostic errors, rather than focusing attention mainly on individual clinical decisions.

## 2. Patients with Non-Specific Complaints: An Interdisciplinary Patient Safety Challenge

Our focus is on older patients (+65 years) with multiple conditions and NSCs, who face increased patient safety risks due to their complex symptoms and chronic care needs. This group of patients is large and growing [1,2,15]. Moreover, their vulnerability to safety risks increases during periods of high workload, when the incidence of patients with NSCs typically rises [16]. This underscores the growing importance of diagnostic work in this area. Thus, managing the risks associated with diagnosing NSCs in this group is crucial. Additionally, multimorbid patients with NSCs are recognized as an emerging and widespread *driver* of ED crowding [15], accentuating an overall impact to patient safety in EDs. Furthermore, these patients have a high risk of discharge/transition without sufficient diagnostics and are prone to being discharged or transitioning with non-diagnosis or undetected conditions, potentially leaving new diagnosis and/or complex care needs to go unnoticed or inadequately addressed [17,18,19,20].

We know that misdiagnosis is often seen with diffuse symptoms, like dizziness and headache, and that patients with NSCs are often triaged as less urgent than patients with disease-specific complaints. We know this can result in higher mortality, longer in-hospital stay, readmissions, and missed diagnosis [12]. However, we know less about the specific in-hospital risks that trigger such outcomes and subsequently how management can improve patient safety through local interventions or implementation of new tailored guidelines and regulatory frameworks. In addition, the overview of non-fatal diagnostic errors and care needs for multimorbid patients is incomplete, perhaps because these errors rarely give rise to compensation from patient insurance schemes or because their consequences are often gradual and therefore cannot be traced back to an error or can occur elsewhere, for instance, in the primary sector after discharge [21]. These risks are difficult to identify with traditional tools such as, e.g., internal audits, and this lack of knowledge impedes targeted efforts. As a consequence, the simultaneous rise of (1) admittances to an ED driven by NSCs registered as generalized weakness or functional impairment and (2) clinical decision-making with an outcome of non-specified diagnosis signals an important and yet currently under-researched patient safety issue related to when, where, and how the risk of diagnostic errors arises in clinical decision-making for multimorbid patients with non-specific chief complaints.

## 3. Emergency Departments as Hotspots for Patient Safety Challenges

Problems pertaining to risks arising from missed, incomplete, or wrong diagnoses in the ED challenge the entire healthcare system, as during the first hours of hospitalization, critical clinical decisions are made. These early decisions set the course for a patient’s treatment trajectory, which may not always provide the necessary care if a diagnostic error occurs. Such errors impact not only immediate patient safety in the ED but also influence subsequent clinical decisions throughout the patient’s care journey. In a specialized healthcare system, a missed opportunity is not easily restored. For instance, in geriatric patients, in whom symptoms are often treated without comprehensive diagnostic testing [22], a missed or wrong diagnosis in the ED can lead to inappropriate treatment plans. This frequently results in the prescription of unnecessary medications and can lead to severe consequences, including medication-related issues [22], prolonged hospital stays, invasive procedures, readmissions, and even permanent disability or death [21,22,23]. Therefore, focusing on reducing diagnostic errors in the ED is crucial for improving patient safety. Furthermore, decisions made in EDs are closely connected to those in primary care and outpatient settings, meaning errors can have cascading effects throughout the healthcare system. This connection complicates efforts to manage and improve patient safety, making it one of the most challenging tasks for researchers and practitioners to prevent delayed, incomplete, or missed diagnoses [5,6]. There are also organizational factors that influence the risks arising from clinical decision-making. One important factor here is that EDs connect the primary and secondary healthcare sectors of health systems, and clinical decisions made in the ED are not made in isolation but must, on some level, acknowledge the capacity of the hospital, as well as of the healthcare system. Lastly, in EDs, healthcare professionals’ diagnostic work is increasingly challenged by a flow culture and by crowding and is complicated by multimorbidity in aging populations and social inequalities in health.

One challenge is how to better qualify and register the ‘messy’ category of NSCs. Factors causing diagnostic errors, and the responsibility of mitigating them, are distributed across various specialized units in the secondary sector and generalists in the primary sector. Specialization in healthcare often leads to a narrow focus that can miss the broader health context of a patient, especially in elderly patients who often manage multiple chronic conditions and take numerous medications. The combination of multimorbidity and polypharmacy can blur the line between symptoms, chronic condition, and medication side effects. For instance, an elderly patient presenting at the ED with symptoms suggestive of delirium could be quickly treated, for instance, for urinary tract infection. and discharged if immediate improvement is observed. This preliminary treatment might delay necessary investigations for underlying conditions like dementia or cancer. Moreover, the common division of labor in healthcare settings can further complicate matters, as subsequent caregivers, especially in primary care, may assume that the secondary care sector has already considered other underlying conditions. They may not immediately recognize the need for further evaluation, believing that the patient has already been extensively investigated for potential medical issues.

When policy makers and healthcare managers develop improvements targeting diagnostics of NSCs, these must acknowledge the patient’s journey through the different parts of the healthcare system, because when consequences of diagnostic errors often occur only after discharge from hospital, they may only be identified in the patient’s own home or by staff in primary sector organizations. Thus, we need to be able to link the causes and effects of diagnostic errors by measures that are operational and meaningful across the implicated parts of the healthcare system, and while the efforts of local managers are important, they must be supported by policies that acknowledge both the clinical and organizational aspects of patient safety. And, because some multimorbid patients with a sufficient high degree of health literacy can act as experts in their own patterns of illnesses and complaints, we also need to integrate their perspectives and experiences as occasions for reflection and adjustment in the organizing and practice of clinical decision-making processes.

## 4. Discussion: Using Clinical and Organizational Research to Improve Patient Safety

The rising numbers of multimorbid patients with NSCs represent a serious challenge to the single-disease paradigm and to traditional diagnostic methods, particularly when acute conditions present where treatment for one condition could potentially exacerbate another condition/comorbidity. This complexity often necessitates a multidisciplinary approach. Diagnosing these patients requires more intricate clinical decision-making to address their diverse treatment and care needs. The traditional boundaries between the symptom-focused approach in primary care and the diagnosis-focused approach in secondary care are changing, and, in some cases, they are being redrawn with new organizational models, e.g., acute care beds in nursing homes and extensions of the physician’s responsibility beyond discharge.

A key element in diagnosing patients with NSCs in EDs with a flow culture involves integrating their symptoms, medical history, and clinical presentation to accurately evaluate the severity of their condition. However, this is fraught with challenges, particularly in acute care settings where older, functionally and cognitively impaired patients often present with complex symptoms. In clinical practice, when the combination of NSC symptoms, patient history, and clinical signs strongly indicates a severe condition, these patients are more likely to undergo thorough examinations, reducing the risk of diagnostic errors. However, a critical issue arises for this patient group when the severity of their condition is not adequately reflected by these factors. Patients whose health problems do not fit this pattern are at a higher risk of diagnostic errors. Such errors can significantly threaten patient safety, with potential repercussions that extend beyond the emergency department and impact broader diagnostic practices. In the organizational context of the ED, these clinical decisions must be carried out under time pressure and with poor help from the diagnostic classification system that by design assumes that diagnoses can be ascertained reliably, fast, and with certainty. Standard methods to study and improve patient safety do not seem optimal either. The patient safety agenda has since the beginning of the century been mostly occupied with safety problems that have been tangible and relatively easily fixed, and diagnostic error has been said to be ‘a major oversight’ [14]. For instance, medication errors have been given much focus, as such are often easily identifiable and categorizable and give way to optimization of the healthcare system through standardization, checklists, or technological fixes [13]. Although diagnostic safety has been more on the patient safety agenda in the past decade [24], the safety methodologies that dominate our health systems still tend to focus on reporting of adverse events, retrospect event analysis (e.g., Root Course Analysis), and system optimization through standardization. These approaches are inadequate for understanding and improving diagnostic processes, which are marked by complexity and inherent uncertainty, often lacking a single identifiable root cause or straightforward solution, especially in cases involving complex multimorbid patients [25]. Moreover, when attempting to pinpoint the causes of diagnostic errors, much emphasis is placed on the cognitive biases of healthcare professionals [26,27], largely ignoring how clinical decision-making is influenced by various organizational and clinical contexts. Furthermore, there remains a significant lack of consensus that necessitates initial clarification and agreement on key terms. Thus, recognizing the inadequacy of relying solely on a single clinical perspective is critical. Instead, adopting an interdisciplinary approach is necessary, as the clinical gaze often focuses too narrowly on diagnosis-specific issues. By prioritizing conceptualization and clarification of concepts before tackling problem-solving, we can deepen our understanding and reduce the frequency of diagnostic errors. Additionally, qualitative, ethnographic methods and insights from organizational research are well suited for in-depth studies of, e.g., how diagnostic work is practiced in situ, in a clinical–organizational micro-system, or how decision-making practices are connected across healthcare organizations and over time in a patient’s trajectory from the home to the ED and onwards. This resonates with alternative trends within patient safety research where the focus is shifting towards understanding and studying safety not only as the absence of error but as a collaborative accomplishment thoroughly dependent on the quality of situated and shared habits and collaborative practices in healthcare [28,29]. Such approaches acknowledge the complexity and situatedness of clinical decision-making and promote a more holistic and effective strategy for enhancing patient safety in the ED and beyond. Thus, we need to include an organizational perspective on patient safety in clinical decision-making processes because missed opportunities for improving diagnosis can arise from a complex interplay of factors stemming from patients, professionals, organizations, and the larger healthcare system. From an organizational perspective, clinical decision-making occurs in a specific local context, where the clinical decision must be made concerning a particular patient. But at the same time, a clinical decision is also formed by the nature and organization of clinical work in, e.g., acute care or elective surgery, by organizational factors such as time pressure, flow culture, or collaboration across professional and organizational boundaries; additionally, the organization and management of the healthcare system in general impact healthcare professionals’ work conditions, for instance, the timeframes for ‘door to doctor’ or national health budgets.

## 5. Conclusions

Clinical decision-making is not a neutral process of interference: it involves professional ethics, understood as the weighing of potentially conflicting managerial, professional, and organizational obligations and norms against one another. Therefore, we suggest that healthcare managers and policy makers adopt a context-sensitive approach to improvements that recognizes the connection between clinical and organizational work features for patient safety. Specifically, we propose a more comprehensive interdisciplinary approach to improvements in patient safety that integrates organizational and clinical research and examines where, when, how, and why risks to patient safety arise in and across different clinical–organizational contexts. Such an approach is crucial for understanding the complexities of diagnostic errors, which often involve multiple, intersecting factors across different domains. By employing existing research on the organization and practice of professional work, expertise, judgment, and decision-making, we can enhance patient safety management, improvement practices, and healthcare policy. This interdisciplinary collaboration is not only vital for recognizing the complexities involved but also for developing more effective interventions to prevent diagnostic errors, ensuring a safer healthcare system for all.

## Data Availability

No new data were created or analyzed in this study. Data sharing is not applicable to this article.
